# Eosinophil Counts in Mucosal Biopsies of the Ileum and Colon: Interobserver Variance Affects Diagnostic Accuracy

**DOI:** 10.1155/2018/2638258

**Published:** 2018-11-04

**Authors:** Florian Hentschel, Anna Franziska Jansen, Marlis Günther, Roland Pauli, Stefan Lüth

**Affiliations:** ^1^Department of Gastroenterology and Hepatology, University Medical Center Brandenburg, Brandenburg, Germany; ^2^Department of Pathology, University Medical Center Brandenburg, Brandenburg, Germany

## Abstract

Primary eosinophilic gastroenteritis and colitis (EGE) is a rare entity with unspecific clinical and endoscopic findings. Validated histopathologic criteria for confirming the diagnosis are lacking, because numeric values for normal or elevated concentrations of eosinophils in mucosal biopsies are varying between observers. To quantify this interobserver variance, we had the same set of 30 slides of eosinophilic-rich mucosal biopsies from the ileum and colon systematically reviewed by a panel of six independent pathologists, each with more than a ten-year experience in the field. Using a highly standardized biopsy and slide preparation protocol, we ruled out any influence by the preparation, the patient, the endoscopist, the endoscopes and calipers used, the sampling site, the fixation and staining method, and the microscopic field sizes. Still, all numeric results differed between pathologists up to a factor greater than 30. Calculated positive or negative diagnosis of EGE differed up to a factor greater than 8. A theoretical incidence for EGE calculated from these numbers differed by a factor greater than 1500. We conclude that eosinophil counts in mucosal biopsies from the lower gastrointestinal tract are subject to a very high interobserver variance. Until further research provides objective and validated methods for standardization, all epidemiologic numbers derived from histopathologic findings may have to be questioned. When diagnosing individual patients with EGE, overall morphologic picture together with clinical and endoscopic findings is more important than numeric eosinophil count.

## 1. Introduction

Noninfectious, or “primary,” eosinophilic gastroenteritis/colitis (EGE / EC) is a rare entity. For clinical and practical reasons, it is distinguished from the better known eosinophilic esophagitis (EoE), although both are suspected to be caused by Th_2_-mediated food hypersensitivity.

Identifying patients with EGE can be demanding. Clinical symptoms like abdominal pain or recurrent diarrhea are unspecific [1–3]. Endoscopic findings are equally uncharacteristic, often mimicking other diseases [4–6].

Diagnosing EGE/EC therefore strongly relies on histopathologic evaluation of mucosal biopsies. But unlike in the esophagus, where diagnostic criteria for EoE are widely agreed upon [7, 8]; there are no such criteria for the ileum or colon. In normal pediatric mucosa, a wide range between 1 and 52 eosinophils per microscopic high power field (HPF) has been reported. Within this range, some observers saw decreasing eosinophil numbers from the cecum to the sigmoid [9, 10], while others showed an increase from the left colon to the rectosigmoid [11]. Values for adults are roughly within the same range, with superimposed seasonal undulations [12]. For North America, a regional increase from north to south has been reported [11, 13], while in Asia there seems to be no difference according to geographic region, race, or sex [14].

Considering this variance, it is not surprising that there is no consensus about the threshold above which the diagnosis of “eosinophilic ileitis/colitis” can be made. Some authors suggest a value as low as at 6 eosinophils per HPF [15], some 15 to 20 [2, 16], some 30 [17], and some 50 [18]. Recently, a differentiated approach was proposed, with upper limits from 50 eosinophils per HPF in the right to 25 the left colon [19].

Because of these varying and contradictory numbers (and considering the comparatively clear situation in the esophagus), many authors suspect an undetected bias in the histopathologic findings. Possible confounders like the variability in microscopic HPF size, selection of fields near or far from lymphoid follicles, and differing criteria for including cells in the count had been discussed [20].

To test these hypotheses, we had the same standardized mucosal biopsies from 10 patients examined by six independent specialists in pathology who were blinded for each other's results. Eosinophils were counted according to a standardized protocol, and results were normalized for the HPF areas of the microscopes used.

## 2. Methods

### 2.1. Patients

We retrospectively examined the histopathologic biopsies of 521 consecutive adult patients who underwent diagnostic ileocolonoscopy in our department from January to December 2017. 84 of them had mucosal biopsies taken because of chronic abdominal pain, recurrent diarrhea, or both. Out of these patients, 10 aroused the clinical or endoscopic suspicion of an elevated eosinophil count in the lower GIT or showed elevated eosinophils in routine histopathologic findings. These were included in the study. Excluded were all patients with inflammatory bowel disease (IBD), collagenous colitis, colonic infections, NSAID treatment, lymphoma, Meniere's disease, and helicobacter pylori infection. All included patients were Caucasians living within < 50 Kilometers from the hospital and prepared for colonoscopy using the same macrogol-based solution (Moviprep, Norgine GmbH, Marburg, Germany).

### 2.2. Biopsies

All patients were examined by one single endoscopist (F.H.). Two biopsies were taken from 3 standardized locations each (terminal ileum, hepatic flexure, rectum) using 2.3mm calipers (MTW Wolfgang Haag KG, Germany) through flexible colonoscopes (Fujinon EC600, Fuji Corp, Japan). Probes were fixated in 4% buffered formaldehyde (R. Langenbrinck GmbH, Emmendingen, Germany) and brought to the pathologist's laboratory. They were then embedded in 10% paraffin wax (Tissue Tek, Sakura Finetek Europe B.V., Netherlands), cut to 4*µ*m slices (Microtome SM2000R/SM2010R, Leica, Germany), and underwent standard H&E staining (Hämalaun Mayer and Hämatoxylin Gill III, Dr. K. Hollborn & Söhne GmbH & Co KG, Germany; Erythrosin, Carl Roth GmbH + Co. KG, Germany) in an automated slide stainer and coverslipper (TCA 44-720, MEDITE GmbH, Germany). After routine histopathologic assessment, they were archived.

For the study, original glass slides of included patients were drawn from the archive, anonymized, and sent to the participating pathologists.

### 2.3. Pathologists and Microscopes

There are six clinical institutes of pathology in the state of Brandenburg. Four of them participated in the study. Mucosal biopsies were examined by six specialists in pathology with at least ten year experience in the field (Box 1), according to standardized counting instructions in German* (for English translation see*Box 2). Microscopes are listed in Table 1. Differing areas of the high power fields (HPF) at 400x magnification were made comparable by a normalization factor, based on current CAP and ITBCC recommendations [23].

### 2.4. Statistics

All data was analyzed using IBM SPSS Statistics 23. If not stated otherwise, eosinophilic numbers are given as mean +/- standard deviation (SD) out of 5 HPF. For metrically scaled, non-Gaussian data, Friedman test was used for more than two paired samples. Cochran Q Test was used for nominal scaled, paired samples.

## 3. Results

**Figure 1:**
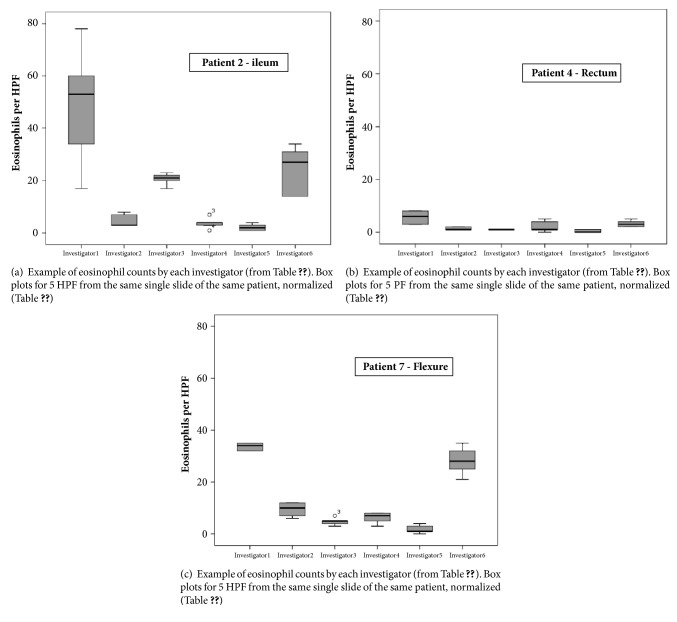


Eosinophil counts in all biopsies differed between investigators, up to a factor > 30. Maximum count was 328 per HPF (mean of 5, hepatic flexure), and minimum count was 0 per HPF (mean of 5, more than one biopsy site). Analyzing each biopsy site for each patient by investigator, differences between the investigators were significant for all biopsy sites (Table 2, Figures 1(a)–1(c)).

Mean eosinophil counts overall differed between the highest and the lowest counting investigator by a factor of 14.5 (29 vs.2).

Intra-individually, each investigator was concordant to his own bias, i.e. the one with the highest counts overall had those highest counts in all but one biopsy, the one with the lowest count had the lowest count in all but three biopsies (Table 2).

**Figure 2:**
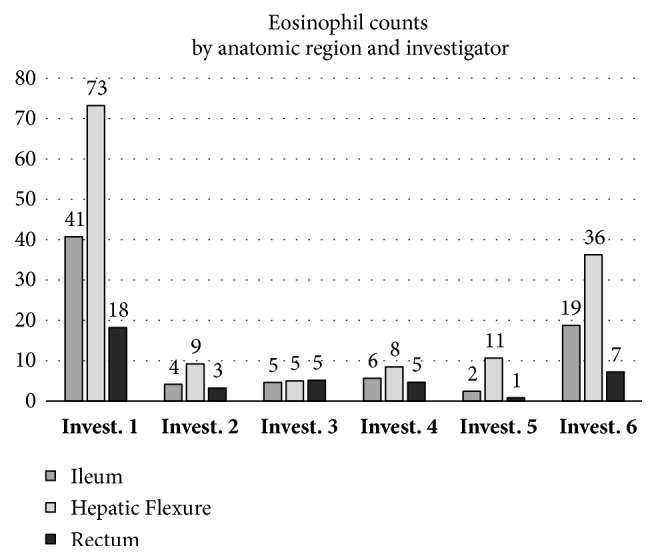
Eosinophil numbers in the ileum, hepatic flexure, and rectum by different investigators (Invest. 1 to 6) examining the same 30 slides according to standardized protocol. Numbers are given as means of 5 HPF from 10 patients = 50 HPF, normalized (Table 2).

Independently of the strong inter interobserver observer variance, each observer found the highest number of eosinophils in the hepatic flexure, with values between 73 eosinophils per HPF for the highest counting investigator (mean of 5 HPF of all patients), and 5 for the lowest. Numbers in the ileum were intermediate in all investigators with values from 41 for the highest counting investigator, and 2 for the lowest. Numbers for the rectum were lowest with numbers from 18 for the highest counting investigator, and 1 for the lowest (Figure 2). Within each investigator, these differences were significant (p < 0.001).

**Figure 3:**
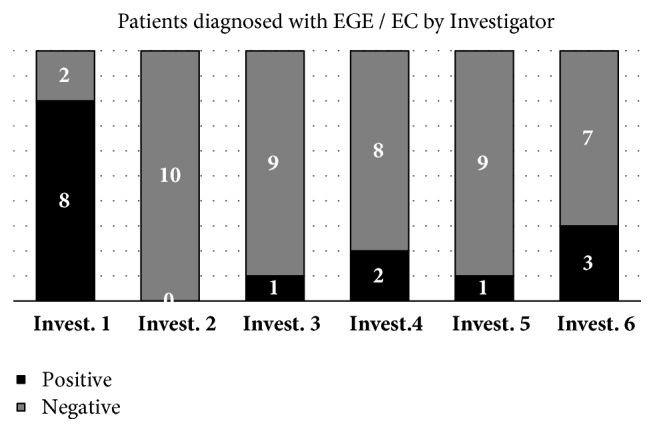
Positive and negative diagnoses for eosinophilic enteritis /colitis by different investigators (Invest. 1 to 6) examining the same 30 slides of 10 patients according to a standardized protocol. Thresholds were Ileum > 20 eosinophiles/HPF, hepatic flexure > 50 eosinophiles/HPF, rectum > 20 eosinophiles/HPF, normalized (Table 2). Results differed significantly (p < 0.05). Changing the thresholds to different values from the literature [9–13] did not change the overall picture (data not shown).

Based on these numbers, we calculated the ratio of positive vs. negative diagnosis for “eosinophilic enterocolitis” for each pathologist, assuming a threshold of 20 eosinophils per HPF in the ileum or rectum and 50 in the hepatic flexure. Results were diverging with observer #1 diagnosing 8 (80%) positive and 2 (20%) negative, while observer #2 diagnosed 0 positive and 10 (100%) negative. Observers #3 and #5 diagnosed 1 (10%) positive and 8 (90%) negative. Observer #4 diagnosed 2 (20%) positive and 8 (80%) negative. Observer #5 diagnosed 1 (10%) positive and 9 (90%) negative, observer #6 diagnosed 3 (30%) positive and 7 (70%) negative (Figure 3). Cochran Q Test showed these differences to be significant (p < 0.05). Changing the thresholds to different values from the literature did not change the overall picture (data not shown).

**Figure 4:**
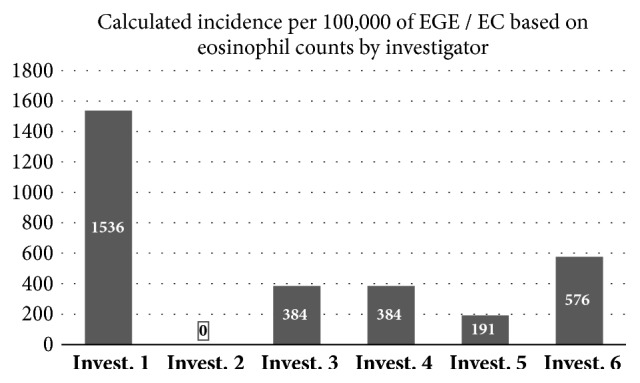
Virtual incidence numbers of eosinophilic enteritis /colitis in adults calculated for each investigator based on 521 patients scanned for the study. Thresholds for positive diagnosis are > 50 Eosinophils per HPF in the hepatic flexure, and > 20 in the ileum or rectum [9–13].

Extrapolating from these numbers to the 521 patients who were initially scanned for the study, we calculated a “theoretical overall incidence” for EGE / EC of 1500 per 100,000 for the highest counting investigator, and < 1 per 100,000 for the lowest counting one (Figure 4).

## 4. Discussion

Eosinophilic diseases of the lower GIT are relatively newly described, incompletely understood, rare, and difficult to detect. Clinical and endoscopic signs are non-specific; histopathologic criteria for mucosal biopsies are lacking. While all authors agree that a certain amount of eosinophils in the ileum and colon is normal, the actual numbers are not known [9–14]. Accordingly, there is no consensus about the limit above which one can safely diagnose an eosinophilic gastroenteritis or enterocolitis. Many authors suggest that the overall morphologic picture together with clinical and endoscopic findings are more important than numeric counts [2, 15, 16, 18, 19, 21]. This is in sharp contrast to the situation in the esophagus, were a number of 16 or more eosinophils per HPF is considered pathognomonic for EoE [7, 8].

Possible explanations for this discrepancy are a lack of clinicopathologic data supporting any particular threshold in the lower GIT, anatomic [9, 10], seasonal [12], genetic or geographic [11, 13] variations in eosinophils, and variations in counting methodology like microscopic field size or criteria of eosinophil counting [20]. Our own suspicion was that numeric eosinophil counts may be observer-dependent.

In this study, we therefore standardized the sampling, processing and examining of 30 slides from ileal, colonic and rectal biopsies from ten Caucasian patients living in the proximity of our center. We then showed the exact same glass slides to six experienced specialists in pathology and asked them to count the eosinophils according to a standardized protocol without knowing the results of each other. Results were normalized to HPF sizes. With this setup, we are confident to have ruled out any influence by the preparation, the patient, the geography, the endoscopist, the endoscopes used, the calipers, biopsy size and number, the topography of the sampling site, the staining method, and the microscopic field sizes. Still, results were strikingly differing.

When discussing this phenomenon with the participating pathologists and gastroenterologists, we did not find an explanation for it right now. One possibility is that the spotty distribution of eosinophils in the lower GIT leads to highly varying concentrations in different HPFs. The error then occurs as early as in the overview, where fields of interest are identified. So, after optical magnification of 400x, every pathologist counts five different regions of the same slide. Further studies, possibly on multiheader microscopes, may be needed to explain these discrepancies. Until then, we can only describe it as extremely high interobserver variance.

Against this background, one may have to rethink some of the facts that are thought to be known about EGE today. Overall prevalence is reported to be about 5 per 100,000 [24, 25], with some observers suggesting it may be higher [17]. One study saw a higher prevalence in male children and female adults [26]: one showed a higher prevalence in Asians [27] and another hinted to a higher incidence in family members of known EGE patients [28]. All of these numbers are derived from histopathologic diagnoses and therefore have to be met with reserve. Extrapolating from our own findings, the true incidence of EGE in our preselected collective could be anywhere between < 1 per 100,000 and 1500 per 100,000.

Since we do not know the exact reason for this phenomenon, we cannot offer a solution right now. Significantly increasing the number of biopsies and HPFs could even out the mean values, but that would be impractical in a real-life clinical situation. Another possibility could be a different staining method to identify and automatically count eosinophils in lower magnification. However, H&E staining that we used is standard for examining mucosal biopsies, and no pathologist reported problems with identifying eosinophils in the slides. So new staining and counting methods would not necessarily be suitable for daily pathologic routine but rather of scientific interest.

## Figures and Tables

**Box 1:**
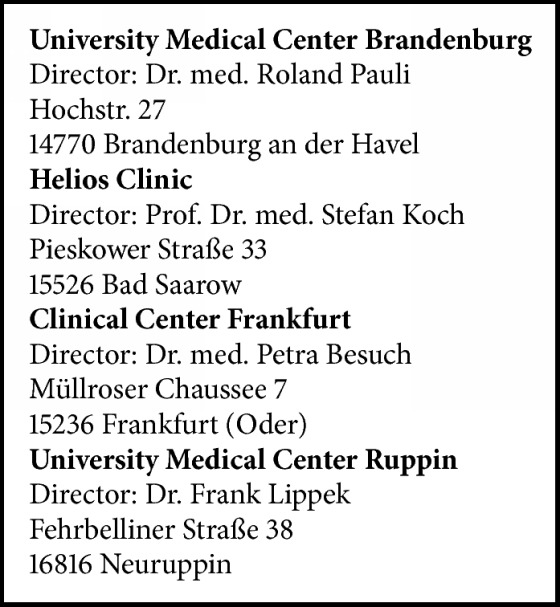
Institutes of Pathology in Brandenburg that participated in the Study.

**Box 2:**
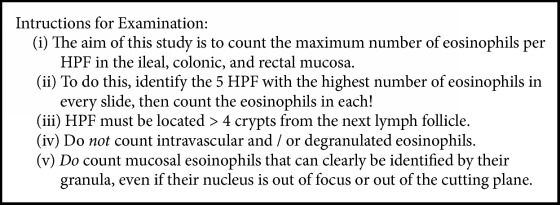
**Instructions for participating pathologists.** These instructions follow known procedures in identifying and counting eosinophils in human mucosa [10, 12, 13, 21, 22].

**Table 1:** Microscopes used by the participating pathologists, area of their high power fields (HPF), and normalization factor [23].

Investigator	Microscope	Area HPF (mm^2^)	Normalization Factor
1	Olympus Bx50 40x/0.65	0.237	1.000

2	Olympus Bx51 40x/0.75	0.344	0.699

3	Olympus Bx51 40x/0.75	0.344	0.699

4	Olympus Bx51 40x/0.75	0.344	0.699

5	Olympus Bx51 (0.55mm) 40x/0.65	0.237	1.000

6	Zeiss Axiolab 40x10/0.65	0.331	0.716

**Table 2:** Eosinophil counts of each pathologist for each anatomic site of each patient. Values shown are mean ± SD from 5 HPF and normalized (Table 1). *∗*Asterisk: Investigator counted 3 HPF as “>100” each, one as 70 and one as 90. *p* = “asymptotic significance” - Friedman test p-value.

		Investigator 1		Investigator 2		Investigator 3		Investigator 4		Investigator 5		Investigator 6		*p *Value
Pat 1	Ileum	**49**	±8,4	**4**	±1,9	**3**	±0,7	**20**	±8,0	**5**	±1,7	**19**	±7,2	< 0.001
	Flexure	**50**	±14,2	**10**	±4,1	**1**	±1,1	**13**	±8,2	**4**	±1,2	**30**	±5,5	= 0.001
	Rectum	**20**	±6,5	**5**	±1,4	**20**	±0,5	**3**	±1,7	**2**	±1,4	**5**	±3,1	< 0.001

Pat 2	Ileum	**48**	±21,1	**5**	±2,7	**7**	±3,1	**4**	±2,6	**2**	±1,2	**24**	±11,7	= 0.001
	Flexure	**328**	±1,4	**25**	±6,4	**13**	±1,4	**(70)**	*∗*	**70**	±6,3	**140**	±19,6	< 0.001
	Rectum	**78**	±12,8	**4**	±1,2	**4**	±4,1	**20**	±14,9	**0**	±0,4	**37**	±18,3	< 0.001

Pat 3	Ileum	**54**	±21,1	**5**	±4,1	**8**	±1,4	**7**	±4,0	**2**	±0,6	**15**	±6,5	< 0.001
	Flexure	**60**	±9,1	**8**	±1,9	**29**	±2,0	**12**	±7,0	**4**	±1,9	**23**	±3,2	< 0.001
	Rectum	**27**	±5,6	**5**	±1,9	**3**	±5,2	**3**	±2,1	**1**	±1,2	**6**	±2,9	< 0.001

Pat 4	Ileum	**11**	±2,7	**1**	±0,5	**6**	±1,2	**4**	±3,0	**3**	±1,7	**4**	±1,3	< 0.05
	Flexure	**30**	±12,8	**6**	±3,0	**1**	±2,0	**7**	±3,6	**6**	±1,6	**22**	±6,0	< 0.05
	Rectum	**6**	±2,2	**1**	±0,5	**3**	±0,4	**2**	±2,7	**0**	±0,5	**3**	±1,5	< 0.05

Pat 5	Ileum	**89**	±14,8	**3**	±1,2	**3**	±1,3	**6**	±1,7	**7**	±3,1	**23**	±9,7	= 0.001
	Flexure	**42**	±13,0	**7**	±1,4	**1**	±1,0	**6**	±2,8	**7**	±2,5	**11**	±1,4	= 0.001
	Rectum	**7**	±3,2	**3**	±0,9	**3**	±0,8	**1**	±1,4	**1**	±0,9	**4**	±1,1	< 0.001

Pat 6	Ileum	**19**	±2,3	**4**	±2,1	**8**	±1,7	**1**	±1,6	**0**	±0,4	**13**	±2,3	< 0.001
	Flexure	**101**	±10,0	**13**	±5,2	**1**	±1,7	**16**	±3,7	**4**	±2,3	**47**	±12,6	< 0.001
	Rectum	**3**	±0,6	**2**	±1,0	**4**	±0,5	**1**	±1,4	**1**	±0,5	**4**	±1,5	= 0.001

Pat 7	Ileum	**68**	±14,4	**10**	±□3,7	**5**	±1,8	**3**	±1,4	**1**	±0,6	**53**	±12,1	= 0.001
	Flexure	**34**	±1,4	**9**	±3,4	**2**	±1,9	**6**	±2,9	**2**	±1,5	**29**	±6,8	< 0.001
	Rectum	**28**	±21,1	**6**	±1,7	**2**	±0,9	**5**	±3,9	**1**	±0,6	**5**	±2,5	= 0.001

Pat 8	Ileum	**31**	±6,7	**5**	±1,0	**3**	±1,0	**4**	±2,3	**2**	±1,1	**18**	±6,7	= 0.001
	Flexure	**34**	±8,7	**2**	±0,6	**1**	±1,5	**6**	±3,2	**4**	±2,0	**19**	±7,4	= 0.001
	Rectum	**7**	±3,2	**3**	±1,0	**2**	±0,7	**6**	±2,6	**1**	±0,5	**5**	±1,3	= 0.001

Pat 9	Ileum	**9**	±2,7	**3**	±1,2	**4**	±0,7	**1**	±1,7	**1**	±0,8	**8**	±2,5	< 0.001
	Flexure	**33**	±8,8	**6**	±1,2	**1**	±1,2	**3**	±2,9	**2**	±0,8	**33**	±6,3	< 0.001
	Rectum	**5**	±0,6	**2**	±1,3	**1**	±0,5	**3**	±2,1	**0**	±0,5	**4**	±1,0	< 0.05

Pat 10	Ileum	**28**	±10,1	**6**	±1,4	**3**	±1,0	**6**	±2,2	**1**	±1,2	**11**	±10,2	< 0.001
	Flexure	**21**	±4,6	**5**	±1,6	**1**	±0,7	**7**	±3,4	**3**	±1,7	**8**	±2,6	<0.001
	Rectum	**2**	±0,4	**1**	±0,5	**3**	±0,5	**2**	±1,0	**0**	±0,4	**1**	±0,7	< 0.05

## Data Availability

Original slides are archived in the Department of Pathology. Counting protocols are available from author Anna Franziska Jansen.

## Abbreviations

CAP: College of American Pathologists
EGID: Eosinophilic gastrointestinal disease(s)
EoE: Eosinophilic esophagitis
EGE: Eosinophilic gastroenteritis/colitis
GIT: Gastrointestinal tract
H&E: Hematoxylin and eosin staining
HPF: High power field
IBD: Inflammatory bowel disease
ITBCC: International Tumor Budding Consensus Conference
NSAID: Nonsteroidal anti-inflammatory drug(s)
SD: Standard deviation.
